# The oligomer modulator anle138b inhibits disease progression in a Parkinson mouse model even with treatment started after disease onset

**DOI:** 10.1007/s00401-014-1265-3

**Published:** 2014-03-11

**Authors:** Johannes Levin, Felix Schmidt, Cathrin Boehm, Catharina Prix, Kai Bötzel, Sergey Ryazanov, Andrei Leonov, Christian Griesinger, Armin Giese

**Affiliations:** 1 Neurologische Klinik, Klinikum der Ludwig-Maximilians-Universität, Munich, Germany; 2 Munich Cluster for Systems Neurology (SyNergy), Munich, Germany; 3 Zentrum für Neuropathologie und Prionforschung, Ludwig-Maximilians-Universität, Feodor-Lynen-Str. 23, 81377 Munich, Germany; 4 NMR-basierte Strukturbiologie, Max-Planck-Institut für biophysikalische Chemie, Göttingen, Germany; 5 DFG Center of Nanoscopy and Molecular Physiology of the Brain (CNMPB), Göttingen, Germany

Parkinson’s disease (PD) is characterized by the deposition of aggregated alpha-synuclein (aSyn). Recent evidence suggests that oligomers formed in the aggregation process constitute the main toxic species causing neurodegeneration. Recently, we reported that the oligomer modulator “anle138b” [3-(1,3-benzodioxol-5-yl)-5-(3-bromophenyl)-1H-pyrazole] is capable of prolonging the survival of prion-infected mice and of various animal models of PD [5]. Anle138b blocked formation and accumulation of aSyn oligomers in the brain, reduced disease-associated motor deficits, and led to prolonged disease-free survival [5]. These findings support the following hypotheses: (i) oligomers formed in the aggregation process of disease-specific proteins, such as prion protein in prion diseases or aSyn in synucleinopathies, constitute the main toxic species involved in neuronal cell death [1–3] and (ii) these oligomers are a suitable target for disease modification [5].

While we could show that anle138b is capable of prolonging the survival of prion-infected mice even after onset of symptoms, it had remained unclear whether anle138b is also effective in animal models of PD when treatment is started secondary to clinical disease onset. The effect of a compound after onset of symptoms is an important prerequisite for its use as a disease-modifying therapy in humans, because clinicians rely on symptoms to establish a diagnosis. Even if early diagnosis from pre-locomotor symptoms such as olfaction problems or aSyn deposits in the gut in combination with imaging or other biomarkers is expected to be available in the future, which would allow earlier initiation of neuroprotective treatment, not all patients may be identified with such a diagnosis. In order to evaluate a potential use of anle138b as a disease-modifying PD therapy, we set out to test the effect of treatment with anle138b after onset of symptoms in a transgenic PD model based on neuronal expression of human A30P-aSyn [4]. Onset of symptoms in the A30P-aSyn mouse model was described by us in the initial publication [5]. In brief, we observed a prodromal disease phase beginning at about 300 days of life with fluctuations in rotarod performance and, beginning at around 350 days of life, a failure to gain body weight [5]. Thus, in the current experiment oral treatment with anle138b was started from the 50th week of life, the time point at which these two clinical signs were present in the previous experiment. The dose of anle138b remained unchanged. Hence, mice received 5 mg anle138b dissolved in 10 μl DMSO mixed with 200 μl peanut butter two times per day. In parallel, a group of transgenic mice was placebo-treated with 10 μl DMSO mixed with 200 μl peanut butter twice daily. Disease progression was monitored by measurements of rotarod performance. As in the previous publication, onset of terminal disease (“disease free survival”) was defined by a decrease in motor performance below mean − 3 SD of the wild-type control mice [5].

First, we analyzed whether the disease progression in the current experiment was comparable to the published data. A comparative analysis by means of a log-rank test of the survival data of the placebo groups of the previous [5] and the current experiment did not show any significant differences between the groups (*p* = 0.49). This is of importance as, in the current experiment with late onset of treatment, only female mice were included to improve uniformity of both experimental groups. As no differences between the experiments were observed, data from female mice in the first experiment (*n* = 6) were included in the data of control mice from the current experiment (*n* = 10). The effect of anle138b was determined by comparative analysis of the survival data of the placebo and the treatment groups (Fig. 1). The log-rank test showed that treatment with anle138b (*n* = 10) beginning at an age of 50 weeks (~350 days) significantly prolonged disease-free survival of the treated transgenic mice (*p* = 0.005). In detail, treatment with anle138b started in week 50 prolonged mean disease-free survival by 59 days (536 vs 477 days). Median survival was prolonged by 57 days. The hazard ratio changed by 0.28 (95 % CI 0.12–0.69). Finally, we compared this treatment effect to the effect of early treatment with anle138b beginning in week 8. In the early treatment experiment, mean survival was prolonged by 66 days (557 vs 491 days) [5]. This analysis indicates that early treatment with anle138b seems only marginally more effective than late treatment. The effect size of the treatment in both experiments was comparable and the log-rank test did not show any significant difference between both groups (*p* = 0.35).

**Fig. 1:**
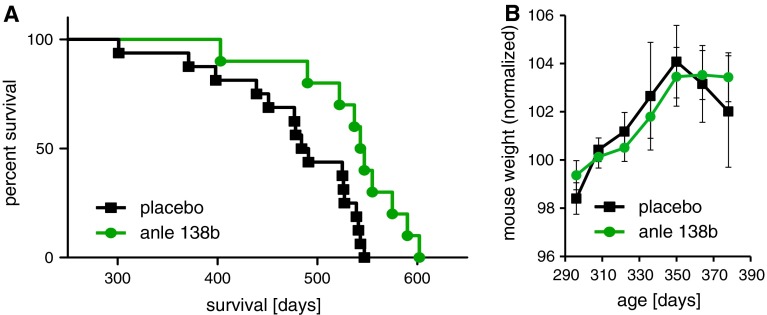
**a** Disease-free survival data of a transgenic PD mouse model based on neuronal expression of human A30P-aSyn treated from week 50 with anle138b compared to placebo. Log-rank analysis revealed a significantly prolonged survival of anle138b-treated A30P-hum-aSyn transgenic mice compared to placebo-treated mice (*p* = 0.005). **b** Mice stop gaining weight at approximately 50 weeks of age, which represents one of the early clinical signs in this mouse model [5]. Body weight was normalized relative to the mean body weight of weeks 42–46

All in all, these data show that treatment with the oligomer modulator anle138b started in the symptomatic disease phase at 50 weeks of life has a significant effect on survival until onset of terminal disease. This is a key prerequisite for disease modification and thus holds promise for a disease-modifying effect of anle138b in PD patients.

## References

[1] Gadad BS, Britton GB, Rao KS (2011). Targeting oligomers in neurodegenerative disorders: lessons from α-synuclein, tau, and amyloid-β peptide. J Alzheimers Dis.

[2] Kostka M, Hogen T, Danzer KM, Levin J, Habeck M, Wirth A, Wagner R, Glabe CG, Finger S, Heinzelmann U, Garidel P, Duan W, Ross CA, Kretzschmar H, Giese A (2008). Single particle characterization of iron-induced pore-forming alpha-synuclein oligomers. J Biol Chem.

[3] Karpinar DP, Balija MB, Kügler S, Opazo F, Rezaei-Ghaleh N, Wender N, Kim HY, Taschenberger G, Falkenburger BH, Heise H, Kumar A, Riedel D, Fichtner L, Voigt A, Braus GH, Giller K, Becker S, Herzig A, Baldus M, Jäckle H, Eimer S, Schulz JB, Griesinger C, Zweckstetter M (2009). Pre-fibrillar alpha-synuclein variants with impaired beta-structure increase neurotoxicity in Parkinson’s disease models. EMBO J.

[4] Neumann M, Kahle PJ, Giasson BI, Ozmen L, Borroni E, Spooren W, Muller V, Odoy S, Fujiwara H, Hasegawa M, Iwatsubo T, Trojanowski JQ, Kretzschmar HA, Haass C (2002). Misfolded proteinase K-resistant hyperphosphorylated alpha-synuclein in aged transgenic mice with locomotor deterioration and in human alpha-synucleinopathies. J Clin Investig.

[5] Wagner J, Ryazanov S, Leonov A, Levin J, Shi S, Schmidt F, Prix C, Pan-Montojo F, Bertsch U, Mitteregger-Kretzschmar G, Geissen M, Eiden M, Leidel F, Hirschberger T, Deeg AA, Krauth JJ, Zinth W, Tavan P, Pilger J, Zweckstetter M, Frank T, Bähr M, Weishaupt J, Uhr M, Urlaub H, Teichmann U, Samwer M, Bötzel K, Groschup M, Kretzschmar H, Griesinger C, Giese A (2013). Anle138b: a novel oligomer modulator for disease-modifying therapy of neurodegenerative diseases such as prion and Parkinson’s disease. Acta Neuropathol.

